# Survival and success of implants in a private periodontal practice: a 10 year retrospective study

**DOI:** 10.1186/s12903-020-01064-z

**Published:** 2020-03-30

**Authors:** Amelie Bäumer, Shirin Toekan, Daniel Saure, Gerd Körner

**Affiliations:** 1 Section of Periodontology, Department of Conservative Dentistry, Clinic for Oral, Dental and Maxillofacial Diseases, University Hospital Heidelberg, Im Neuenheimer Feld 400, 69120 Heidelberg, Germany; 2 Private Practice, Niedernstrasse 16, 33602 Bielefeld, Germany; 3 Private Practice, Dortmund, Germany; 4 Institute of Medical Biometry and Informatics, University of Heidelberg, 69120 Heidelberg, Germany

**Keywords:** Implants, Survival, Mucositis, Peri-implantitis, XiVE

## Abstract

**Background:**

To assess long-term results of implants (XiVE/Frialit-2 Synchro) in a private periodontal practice according to survival and success rates (biological and technical complications) and to detect possible influencing factors, retrospectively.

**Methods:**

Implant placement of at least one implant took place 10 years ±6 months before clinical and radiographic re-examination. Incidence of implant loss as main and incidence of mucositis/ peri-implantitis as secondary outcome were detected. Also, patient-related and implant-related influencing factors were determined by regression analyses.

**Results:**

100 patients (59.0% female) with 242 implants were included into analysis. Survival rate was 94.0% (XiVE: 97.7%; Frialit-2-Synchro: 66.7%). Mucositis was found in 77.6% of all patients, moderate/severe peri-implantitis in 16.3%. In logistic regression analyses statistically significant influencing factors for implant loss was implant type (*p* < 0.001), for mucositis a wider implant diameter (*p* = 0.0438) and a high modified Plaque Index (*p* = 0.0253), for peri-implantits number of implants per patient (*p* = 0.0075) and a wider implant diameter (*p* = 0.0079). Technical complications were found in 47 implants (19.4%).

**Conclusions:**

XiVE implants showed a high survival rate over a 10-year follow-up, on the other hand Frialit-2 Synchro implants had worse survival rates. Success rates regarding biological complications are in line with other implant systems.

## Background

Long-term data regarding survival and success rates over periods of 10 years or more are available for different implant systems [1–4]. However, just one long-term study over 10 years is published for the implant system XiVE S Cellplus (Fa. Dentsply Sirona Implants, Mannheim, Germany) with an implant survival rate of 83.0% [5], another one over a shorter observation period of 7.5 years [6]. For the implant system Frialit-2-Synchro (Fa. Dentsply Sirona Implants, Mannheim, Germany) similar survival rates of 87% over a period of 4.5 years can be found [7] as well as stable bone changes over 10 years [8]. However, long-term data on these implant systems are still rare.

Also, the incidence of peri-implant mucositis and peri-implantitis is still not clear because of different definitions in the literature [9, 10]. A review by Derks & Tomasi (2015) stated the incidence of peri-implant mucositis with a wide range of 19–65% (weighted mean prevalence of 43%) and of peri-implantitis with 1–47% (22%, respectively). Furthermore, they found that extent and severity of the disease were rarely reported.

Several influencing factors for peri-implant mucositis and peri-implantitis are also described. For the development of peri-implant mucositis the factors plaque accumulation, residual cement excess or smoking seems to have an impact [11]. For peri-implantitis risk factors such as smoking [12–14], a history of periodontitis [4, 15, 16], plaque accumulation [17], non-compliance to recall [18], residual excess cement [19] or number of implants [15] could be detected. But there might be more influencing factors for these diseases than assessed so far, e.g. the width of keratinized gingiva.

Due to the few long-term data for the implant systems XiVE and Frialit-2 Synchro, the aim of this study was to assess long-term data of biological (survival rates, mucositits, periimplantitis) and technical complications of these implants placed in patients in a private periodontal practice. It was supposed that these implants show similar results like others do in a periodontally compromised but treated clientel. Furthermore, potential influencing factors for implant loss, peri-implant mucositits and peri-implantitis should be determined.

## Methods

The study was performed in accordance with the Declaration of Helsinki 1975, as revised in 2008, and was approved by the Institutional Review Board for Human Studies of the Medical Faculty of Heidelberg University (Application# S-210/2013). All patients were informed about possible risks and benefits as well as the procedures of the study and all gave written informed consent at the re-examination.

The presented study has a retrospective design combined with a prospective long-term re-examination.

### Study population

All patients were treated in a private practice from 2003 to 2006 by implant placement of at least one XIVE S Cellplus or Frialit-2-Synchro Implant (Fa. Dentsply Sirona Implants, Mannheim, Germany) by one surgeon (GK). All patients received at least one session of individual hygiene before implants were placed. When a periodontitis was detected a complete active periodontal therapy (APT) was conducted before. Smokers received implant treatment if the maximum daily dose did not exceed 10 cigarettes/day. After implant placement all patients were incorporated into a recall program. This contained re-instruction and re-motivation to an effective individual plaque control, professional mechanical plaque removal and once a year obtaining a dental and periodontal status. Sites exhibiting periodontal pocket depth (PPD) of 4 mm plus bleeding on probing (BOP) and sites with PPD ≥ 5 mm were scaled subgingivally [20, 21].

All treated patients were invited to a re-examination 10 years ±6 months after implant placement until 100 patients could be included when fulfilling the following inclusion criteria:

- Available radiograph at implant placement (+ 3 months) and/or time of inserting the implant-supported prosthetic (orthopantomogramm or x-ray)
- Available attachment level or panoramic radiograph/complete x-ray status to classify patient’s periodontal diagnosis at baseline
- ≥ 18 years at re-examination
- Non-pregnant or breastfeeding
- Partially edentulous dentition
- At least one XIVE S Cellplus or Frialit-2-Synchro Implant (all patients with Biomet Implants were excluded due to the low number of these patients (*n* = 3)).

### Surgical and prosthetic procedures

Data on time for healing after extraction was recorded. The surgical procedure included bone augmentation, if necessary. One week after implant insertion the sutures were removed. In cases without bone augmentation or only minor augmentations loading of the implants was conduced about 3 months after implantation, in cases of major augmentation (e.g. external sinus lift, block augmentation) loading time was about 4–5 months. Fixed supra-structures were mostly cemented with a temporary cement (TempBond®). In case of decementation, Rely X® was taken as cement. Just few supra-structures were screwed.

### Clinical examination

Complete clinical re-examinations were performed by one independent and calibrated examiner (AB) from November 2013 to May 2016. They included:

- Medical history
- Familial history regarding periodontitis
- Self-reported comprehensive smoking history, whereby patients were categorized as current, former and non-smokers [22] as well as measurement of carbon monoxid via Compact Smokerlyzer® (Fa. Bedfont Scientific Ltd., UK)
- Questionnaire on smoking at baseline (current, former and non-smoker)
- Self-reported educational status and classification into three groups: low (primary school), moderate (intermediate secondary education, apprenticeship) or high (upper secondary education)
- Questionnaire on complications with implant-supported restorations during the last 10 years: none, major complications (implant fracture, loss of supraconstruction), medium complications (abutmend fracture, veener or framework fracture, phonetic complications), minor complications (abutment/screw loosening, de-bonding, loss of retention, minor chipping)
- Dental status
- Periodontal status: probing pocket depth (PPD) and vertical attachment levels (CAL-V) to the nearest 1 mm using a manual periodontal probe (PCPUNC 15; HuFriedy, Chicago, IL, USA) at six sites per tooth/implant, bleeding on probing (BOP) and suppuration on probing (SUP), assessment of furcation involvement [23] and mobility
- Modified Gingival Index (mGI) and modified plaque index (mPII) at all implants ([24])
- Width of keratinized mucosa at six sites per implant in mm (in the maxilla just three sites due to the masticatory palatal mucosa)
- Digital x-ray (periapical radiographs) of all implants
- Evaluation of the implant-supported restoration at each implant regarding technical complications

### Patients’ charts

Retrospective evaluation of patients’ charts was accomplished by two examiners (ST, AB) independently and included:

- Baseline periodontal diagnosis according to the classification from 1999 [25] retrospectively on the basis of the baseline examinations (dental and periodontal status, radiographic examination)
- Periodontal treatment before implant placement (none, non-surgical, surgical)
- Compliance to the recall program: a frequency of at least two visits per year was recommended. When extending the recall interval once over 100% (i.e. returning after 13 months for recall) the patient was non-compliant.
- Recurrence of periodontal disease: at re-examination percentage of sites with PPD ≥ 5 mm was detected. A recurrence of active periodontal disease was considered, if more than 30% of a patient’s teeth showed PPD of ≥5 mm at re-examination [21]
- Type, length and diameter of implants
- Time of implant placement
- Augmentation of soft/hard tissues and time of augmentation
- Form of implant healing (submerged or non-submerged)

### Radiographic analysis and assessment of bone loss

Bone loss was calculated by comparing baseline radiographs with radiographs at re-examination. The following distances were measured at the mesial and distal aspect of the implant by using a computer program (VixWin Platinum Version 1.4, Fa. Gendex, Hatfiels, USA) under standardized conditions in a darkened room by two examiner (ST, AB):

- Implant shoulder to limbus alveolaris or if present to bony defect
- Implant length (‘apical-coronal’ length).

The implant length reported by the manufacturer was used for the calibration of the distances. The largest value was taken as the extent of bone loss. As proposed a measurement error of 0.5 mm was included [26]).

### Definition of biological complications

According to Derks et al. (2016) mucositis was defined as presence of BOP/suppuration but no detectable bone loss. Peri-implantitis was defined as presence of bone loss of > 0.5 mm with/without BOP/SUP and a moderate/severe peri-implantitis was stated when bone loss reached > 2.0 mm. Implant survival was given when the implant was not lost during the last 10 years.

### Statistical analysis

All data were entered into two separate data files (Excel version 2003, Microsoft Corporation, Redmond, WA, USA) by two investigators (ST, AB). These data were compared thereafter. All differing entries were double-checked by means of comparison with the original patients’ charts.

Primary outcome was survival of implants, secondary outcome implant success (no mucositis, no peri-implantitis, no technical failures).

Descriptive statistical analysis with rates for qualitative characteristics and with mean, standard deviation, median, minimum and maximum for quantitative outcome was performed using R 3.2.2 (R Foundation for Statistical Computing, Vienna, Austria, www.r-project.com). This software was also used for logistic regressions with either implant loss or mucositis or peri-implantitis or severe peri-implantitis as dependent variable on both patient and implant level. As independent factors pre-defined variables were included without variable selection and the significance level was chosen to be 0.05, which was not adjusted for multiple testing, also because of the descriptive nature of this study. A χ^2^-test was conducted to test for a difference between smokers and non-smokers according to smokerlyzer. Spearman correlation was used to correlate the width of keratinized gingiva with peri-implantitis, mucositis, BOP, mPII and mGI.

## Results

### Study population data

**Fig. 1:**
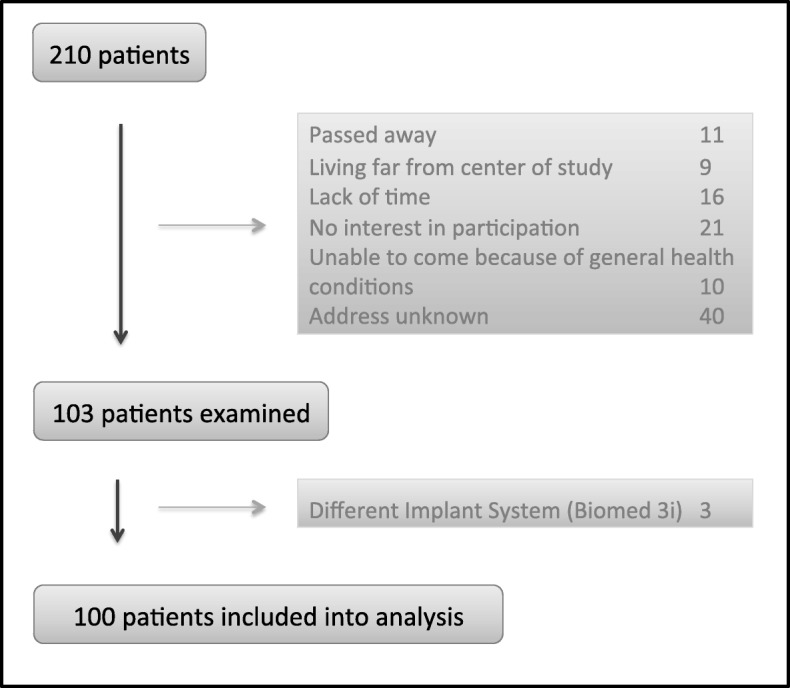
Recruitment of patients included in the study

210 patients received an implant treatment during 2003 and 2006 and fulfilled the inclusion criteria, 103 of them could be reexamined (responder rate of 49.1%). In the analysis 100 patients aged 28–86 years (mean 63.8 years, SD 10.3 years) were included 10 years (range 9.5–10.7 years, SD 0.31 years) after implant setting. The reason for exclusion is given in Fig. 1.

59.0% were female and most of the patients had a high educational level (64.0%; 26.0% moderate, 5.0% low, 7.0% unknown). At implant placement 25.0% were smoker, at reexamination still 10.0% (former smoker 45.0%, never smoker 45.0%). A correlation between low smokerlyzer levels and by questionnaire-defined non-smokers could be stated (*p* < 0.001). At baseline 27.0% of all patients had no periodontitis, 54.0% a chronic and 16.0% an aggressive periodontitis. Before implant placement all patients with periodontitis were treated non-surgically, 52.0% afterwards also surgically. 60.0% were compliant to the SPT. At reexamination 5.0% showed a recurrence of periodontitis.

### Implants

A total of 242 implants could be included. 212 Xive S Cellplus implants (87.6%) in 88 patients (88.0%), 30 Frialit-2-Synchro implants in 12, respectively. Number of implants per patient is given in Table 1. Time of implant placement, form of augmentation, healing as well as length and diameter of implants is presented in Table 2. After implant placement 8.7% of all implants needed peri-implant therapy (5.0% non-surgical, 3.7% surgical). At re-examination data regarding bone loss, PPD, width of keratinized gingiva, mPII, mGI and BOP were collected and are given in Table 2.

**Table 1:** Distribution of implants per patient

Number of implants per patient	Amount of patients
	*n* = 100
1 Implant	35 (35.0%)
2 impants	29 (29.0%)
3 implants	15 (15.0%)
4 implants	11 (11.0%)
5 implants	5 (5.0%)
6 implants	3 (3.0%)
8 implants	1 (1.0%)
9 implants	1 (1.0%)

**Table 2:** Implant data at baseline (a) and at re-examination (b)

a					
	Total	XiVE	Frialit-2 Synchro	Implant lost	Implant in situ
Baseline data	*n* = 242	*n* = 212	*n* = 30	*n* = 9	*n* = 233
Implanttyp					
XIVE S Cellplus	212 (87.6%)			2 (0.9%)	210 (99.1%)
Frialit-2-Synchro	30 (12.4%)			7 (23.3%)	23 (76.7%)
Implant diametre					
3 mm	3 (1.3%)	3 (100%)	0 (0%)	0 (0%)	3 (100%)
3.4 mm	28 (11.6%)	24 (85.7%)	4 (14.3%)	4 (14.3%)	24 (85.7%)
3.8 mm	111 (45.9%)	94 (84.7%)	17 (15.3%)	2 (1.8%)	109 (98.2%)
4.5 mm	88 (36.4%)	80 (90.9%)	8 (9.1%)	2 (2.3%)	86 (97.7%)
5.5 mm	12 (5.0%)	11 (91.7%)	1 (8.3%)	1 (8.3%)	11 (91.7%)
Implant lenght					
8 mm	2 (0.8%)	2 (100%)	0 (0%)	0 (0%)	2 (100%)
9.5 mm	10 (4.1%)	10 (100%)	0 (0%)	1 (10.0%)	9 (90.0%)
10 mm	6 (2.5%)	0 (0%)	6 (100%)	2 (33.3%)	4 (66.7%)
11 mm	40 (16.5%)	39 (97.5%)	1 (2.5%)	0 (0%)	40 (100%)
13 mm	138 (57.0%)	122 (88.4%)	16 (11.6%)	1 (0.7%)	137 (99.3%)
15 mm	46 (19.0%)	39 (84.8%)	7 (15.2%)	5 (10.9%)	41 (89.1%)
Implant position (Jaw)					
Maxilla	136 (56.2%)	121 (89.0%)	15 (11.0%)	1 (0.7%)	135 (99.3%)
Mandible	106 (43.8%)	91 (85.9%)	15 (14.1%)	8 (7.5%)	98 (92.5%)
Implant Position					
Anterior	56 (23.1%)	42 (75.0%)	14 (25.0%)	6 (10.7%)	50 (89.3%)
Posterior	186 (76.9%)	170 (91.4%)	16 (8.6%)	3 (1.6%)	183 (98.4%)
Time of Implant placement					
time of tooth extraction not known	21 (8.7%)	17 (81.0%)	4 (19.0%)	0 (0%)	21 (100%)
immediate implant	23 (9.5%)	18 (78.3%)	5 (21.7%)	4 (17.4%)	19 (82.6%)
delayed immediate implant	1 (0.4%)	1 (100%)	0 (0%)	0 (0%)	1 (100%)
6–12-weeks afer extraction	18 (7.4%)	15 (83.3%)	3 (16.7%)	2 (11.1%)	16 (88.9%)
> 12 weeks to < 6 months	22 (9.1%)	19 (86.4%)	3 (13.6%)	0 (0%)	22 (100%)
≥ 6 Monate	157 (64.9%)	142 (90.5%)	15 (9.5%)	3 (1.9%)	154 (98.1%)
Augmentation					
none	51 (21.1%)	45 (88.2%)	6 (11.8%)	1 (2.0%)	50 (98.0%)
internal sinus lift	9 (3.7%)	9 (100%)	0 (0%)	0 (0%)	9 (100%)
external sinus lift	54 (22.4%)	50 (92.6%)	4 (7.4%)	0 (0%)	54 (100%)
bone substitute/membran	111 (45.9%)	97 (87.4%)	14 (12.6%)	4 (3.6%)	107 (96.4%)
block augmentation	9 (3.7%)	8 (88.9%)	1 (11.1%)	0 (0%)	9 (100%)
soft tissue	3 (1.3%)	2 (66.7%)	1 (33.3%)	0 (0%)	3 (100%)
distraction osteogeneses	5 (2.1%)	1 (20.0%)	4 (80.0%)	4 (80.0%)	1 (20.0%)
Implant healing (*n* = 234)					
submerged	176 (75.2%)	153 (86.9%)	23 (13.1%)	1 (0.6%)	175 (99.4%)
non-submerged	58 (24.8%)	57 (98.3%)	1 (1.7%)	8 (13.8%)	50 (86.2%)
Connection implant/suprastructure					
cemented	227 (93.8%)	210 (92.5%)	17 (7.5%)	0 (0%)	227 (100%)
screwed	6 (2.5%)	0 (0%)	6 (100%)	1 (16.7%)	5 (93.3%)
b					
Reexamination data	Total *n* = 233				
PPD (mean per implant)	3.16 mm ± 1.02 (range 1.00–8.00 mm)				
Bone loss (maximum per implant)	0.78 mm ± 1.41 (range 0.00–6.10 mm)				
< 1 mm	63.3% of all implants				
1 mm - < 2 mm	16.5%				
2 mm - < 3 mm	9.4%				
3 mm - < 4 mm	4.7%				
≥ 4 mm	6.1%				
BOP (at least one positive site per implant)					
positive	202 (86.7%)				
negative	31 (13.3%)				
Width of keratinized gingiva					
0 mm	31 (13.3%)				
0.5 mm	1 (0.4%)				
1.0 mm	32 (13.7%)				
1.5 mm	1 (0.4%)				
2.0 mm	70 (30.0%)				
3.0 mm	48 (20.6%)				
3.5 mm	1 (0.4%)				
4.0 mm	27 (11.6%)				
5.0 mm	13 (5.6%)				
6.0 mm	9 (3.9%)				
mod. GI (maximum per implant) according to Mombelli et al. (1987)					
0	130 (55.8%)				
1	62 (26.6%)				
2	32 (13.7%)				
3	9 (3.9%)				
mod. PI (maximum per implant)					
0	54 (23.2%)				
1	71 (30.5%)				
2	85 (36.5%)				
3	23 (9.9%)				

*PPD* periodontal pocket depth, *mod. GI* modified Gingiva Index, *mod. PI* modified Plaque Index, *BOP* bleeding onb probing

### Biological complications

#### Survival

On patient-level survival rate of implants was 94.0% (XiVE: 97.7%; Frialit-2-Synchro: 66.7%). Six patients lost at least one implant (five patients one implant, one patient four implants), five of them received more than one implant. Therefore, 98 patients are included into data collected at re-examination.

The possible influencing factors for implant loss such as age, sex, smoking at implant placement, periodontal diagnosis at re-examination, compliance, number of implants per patient, implant type and recurrence of periodontits were assessed in a regression analysis. A statistically significant influence could be found for the factors ‘implant type’ (*p* < 0.001): Frialit-2-Synchro implants were lost more often than XiVE implants (Table 4a).

On implant-level nine implants were lost (survival rate 96.3%), seven were Frialit-2-Synchro implants and two XiVE. Most of them (8 implants, 3.3%) were lost in the healing phase, only one implant (0.4%) was lost due to peri-implantitis and was removed 8 years after implant placement. Therefore, data of 233 implants is given in the re-examination data.

There were too few events of implant loss, therefore, a regression analysis on implant-level was not possible.

#### Success

Mucositis could be found in 77.6% of all patients and at 61.4% of all implants (patients/implants with peri-implantitis included). None of the factors seemed to have impact on the incidence of mucositis on patient-level. As influencing factors on implant-level the regression analysis determined a wider implant diameter (*p* = 0.0438) and a higher PI (*p* = 0.0253) as statistically significant (Table 4a). No statistical significance could be found for the factors width of keratinized gingiva, implant length, augmentation of soft/hard tissue and implant healing.

As defined by Derks et al. (2016), on patient-level a peri-implantitis was detected in 54.1% of all cases and a moderate/severe form of peri-implantitis in 16.3%. Regression analysis for moderate/severe peri-implantitis detected a higher number of implants as influencing factor (*p* = 0.0075) (Table 3b). On implant-level, a peri-implantitis could be seen in 41.2% of all implants and a moderate/severe peri-implantitis in 10.3%. As on mucositis a wider implant diameter seemed to have a statistically significant influence on the incidence of a moderate/severe peri-implantitis (*p* = 0.0079) (Table 4b).

**Table 3:** Regression analyses regarding patient-related factors

	Estimate	SE	*t*	*P*
a. Regression analysis: *implant loss* in relation to patient-related factors				
(Intercept)	−0.2056	0.1823	−1.128	0.262
Age (1 year)	0.0025	0.0028	0.922	0.359
Sex (female)	0.0482	0.0577	0.835	0.406
Smoking at implant placement	− 0.0513	0.0651	− 0.789	0.432
Periodontal diagnosis at baseline	0.0077	0.0183	0.418	0.677
Compliance	0.0641	0.0587	1.093	0.277
Counts of implants in each patient	0.0021	0.0179	0.118	0.907
Implant type	0.3755	0.0879	4.274	< 0.001
Recurrence of periodontitis at reexamination	− 0.0671	0.1305	− 0.514	0.609
b. Regression analysis: incidence of *moderate/severe peri-implantitis* in relation to patient-related factors				
(Intercept)	−0.1003	0.2518	− 0.398	0.6913
Age (1 year)	0.0015	0.0038	0.395	0.6939
Sex (female)	−0.0495	0.0798	−0.621	0.5363
Smoking at implant placement	0.0932	0.0905	1.029	0.3061
Periodontal diagnosis at baseline	0.0048	0.0250	0.190	0.8497
Compliance	0.0370	0.0803	0.461	0.6459
Counts of implants in each patient	0.0673	0.0246	2.734	0.0075
Implant type	−0.1098	0.1252	−0.877	0.3829
Recurrence of periodontitis at reexamination	−0.1087	0.1777	−0.612	0.5422

Dependent variable: implant loss after 10 years; *n* = 100

Dependent variable: moderate/severe peri-implantitis after 10 years; *n* = 100

**Table 4:** Regression analyses regarding implant-related factors

	Estimate	SE	*t*	*P*
a. Regression analysis: incidence of *mucositis* in relation to implant-related factors				
(Intercept)	−6.3125	3.0371	−2.0785	0.0377
Width of keratinized gingiva	0.2260	0.1493	1.513	0.1302
Implant length	0.0923	0.1580	0.5846	0.5588
Implant diametre	0.9410	0.4668	2.0160	0.0438
Augmentation of hard/soft tissue	0.1830	0.1917	0.9549	0.3396
Implant healing (submerged)	0.6991	0.6353	1.1003	0.2712
Implant type	−0.2532	0.8666	−0.2922	0.7701
Plaque Index	0.6068	0.2713	2.2367	0.0253
b. Regression analysis: incidence of *moderate/severe peri-implantitis* in relation to implant-related factors				
(Intercept)	4.3652	15.3687	0.2840	0.7764
Width of keratinized gingiva	−0.8289	0.4598	−1.8028	0.0714
Implant length	0.6268	0.9761	0.6422	0.5208
Implant diametre	−4.9021	1.8460	−2.6555	0.0079
Augmentation of hard/soft tissue	−0.2617	1.3961	− 0.1875	0.8513
Implant healing (submerged)	−1.1041	6.0597	−0.1822	0.8554
Implant type	−16.0250	10.8131	−1.4820	0.1383
Plaque Index	1.0647	0.8470	1.2570	0.2087

Dependent variable: mucositis after 10 years; *n* = 233

#### Width of keratinized gingiva

31 implants (13.3%) presented no keratinized gingiva. The width of keratinized gingiva was correlated with peri-implantitis, mucositis, BOP, mPII and mGI, but showed only statistical significance with the presence of BOP (*p* = 0.045, *r* = 0.132). For the other factors no significance could be found, although mucositis almost proved to be significant (*p* = 0.075, *r* = 0.177).

### Technical complications

Most implants were supported by single crowns (55.6%). 25.6% of implants served as bridge anchor for implant-supported fixed dental prostheses (FDP), 17.5% as anchor for implant-supported removable prostheses, 1.3% as bridge anchor for combined tooth-implant-supported FDPs. Most implant-supported crowns were cemented (97.4%).

Technical complications were found in 47 implants (19.4%). Abutment/screw loosening occurred in 13 cases and was the most common complication (5.3%) (Table 5). No implant fracture took place.

**Table 5:** Technical complications

	Implants
	*n* = 242
No complication	195 (80.6%)
Complications (more than one per implant possible)	47 (19.4%)
*a) Major complications*	
Implant fracture	0 (0%)
Loss of supraconstruction	4 (1.7%)
*b) Medium complications*	
Abutmend fracture	1 (0.4%)
Veneer or framwork fracture	10 (4.0%)
Phonetic complications	0 (0%)
*c) Minor complications*	
Abutmend/screw loosening	13 (5.3%)
De-bonding	0 (0%)
Loss of retention	3 (1.2%)
Minor chipping	10 (4.0%)

## Discussion

Long-term data over 10 years regarding survival and success rates of XiVE S and Frialit-2 Synchro implants are rare. Therefore, the aim of this retrospective study was to assess survival and success rates of these implant systems over a follow-up period of 10 years. For XiVE implants similar results as for many other implant systems could be found, the survival rates for Frialit-2 Synchro implants was noticeably lower.

### Biological complications

The observed *survival rate* over 10 years of 94.0% (97.7% for XiVE-implants) on patient-level presents comparable data to another long-term study by Degidi et al. (2016) of 93.0%. Similar survival rates can be found for other implant systems over the same follow-up period [3, 4, 27]. Most implants (8 of 9) were lost in the early healing phase prior the implant-supported restoration was connected (5.0% of patients, 3.3% of implants), just one in the late phase due to peri-implantitis (1.0% of patients, 0.4% of implants). Also, mostly Frialit-2-Synchro implants were lost. In a large study on early and late implant loss Derks et al. (2015) detected comparable rates of early loss in 4.4% of patients (1.4% of implants), but a higher late implant loss in 4.2% of patients (1.4% of implants) 9 years after implant placement. Overall, implant survival rates were in accordance with the long-term data over a follow-up period of 10 years published so far.

In our analysis ‘implant type’ (Frialit-2-Synchro) was the sole statistically significant influencing factor for implant loss as described by Derks et al. (2015), who detected ‘implant brand’ as an influencing factor. All other potential influencing factors such as smoking or history of periodontitis had no impact, as supported by several other studies [2, 4, 5, 16]. This might be due to the low number of lost implants, wherefore detection of influencing factors on implant-level was not possible. Another reason could be that all periodontally compromised patients had received a periodontal treatment of high quality in the private periodontal practice prior to implant placement and afterwards showed a high compliance to the supportive implant therapy.

The high implant loss rate of Frialit implants might be biased due to the relatively small number of observed implants and that solely one patient lost 4 of those implants in the early healing phase.

For comparison of *success rates* the definition of mucositits (BOP/suppuration without bone loss) and peri-implantitis (BOP/suppuration and bone loss > 0.5 mm; moderate/severe peri-implantitis: BOP/suppuration and bone loss > 2.0 mm, respectively) were chosen from a large study from Sweden [15]. Interestingly, our incidences for moderate/severe peri-implantitis (16.3% of patients, 10.3% of implants) were quite in accordance with the data published by Derks et al. (2016) for moderate/severe peri-implantitis (14.5% of patients, 8.0% of implants). Also, our results are reflected in the data published by Mombelli et al. (2012), which provided an incidence of peri-implantitis of 20% on patient-level and 10% of implant-level as well as by the one by Roos-Jansaker et al. (2006) of 16%. The number of implants per patient could be established as an influencing factor for moderate/severe peri-implantitis, which Derks et al. (2016) also found in their study. Furthermore, on implant-level a wider implant diameter was detected as influencing factor for peri-implantitis. All other risk factors proposed in many other studies such as smoking, history of periodontitis, plaque accumulation or compliance to recall [4, 15, 16, 18, 27] reached no statistically significant influence in our analysis. Also, the often discussed factor ‘width of keratinized gingiva’ could not been detected as influencing factor for peri-implantitis in regression analyses, even if there was a slight correlation between width of keratinized gingiva and higher mod. PI at the implant. Also, higher mod. PI as well as a wider implant diameter was associated with peri-implant mucositis. That plaque accumulation results in peri-implant mucositis was often stated by different authors [17, 27–29] and is underlined by our data.

### Technical complications

There still seems to exist a substantial lack of well-performed longitudinal reports on implant-supported restoration over an observation period of ten or more years [30]. In our studies, technical complications occurred in about 20% of all implants, comparable to the 16-years results by Simonis et al. (2010) with 31.1%. The most common complication over this 10 years follow-up was abutment/screw loosening, which occurred in 5.3% of all implants. Chipping could be observed in 4.0% of all implant-supported restorations and is comparable with chipping of the veneering material of fixed dental prosthesis (4.1%) in an actual review assessed over 5- and 10-years [31].

### Limitations

This analysis has some limitations (retrospective design, limited number of patients and implants with few events regarding implant loss) and could potentially be biased because of a relatively homogenous clientele of mostly highly educated patients, a high quality standard of dental/periodontal care in a specialized periodontal practice and the form of recruitment (few implants of the brand Frialit-2-Synchro compared to XiVE implants). On the other hand, the enrolled patients showed a high heterogeneity regarding age (between 28 and 86 years), smoking status and history of periodontitis. Also, a wide heterogeneity regarding form and extent of augmentation, point of time of implantation after extraction or variations in type of implant loading could have influenced the results. Likewise, the inconsistent distribution of insertion of the two different implant types (XiVE or Frialit-2 Synchro) could have biased the data. Also, the responder rate of about 50% could lead to a selection bias (e.g. patients were not willing to participate possibly due to dissatisfaction/problems with their implants).

## Conclusions

In this retrospective long-term study over 10 years on XiVE S Cellplus/Frialit-2-Synchro implants survival and success rates are assessed. This data show high survival rates of implants and comparable incidences of peri-implant mucositits and peri-implantitis for XiVE implants compared to other implant systems; for the system Frialit-2 Synchro the result were worse. However, the source of bias of this study has to be kept in mind due to variable aspects. Major technical complications occurred rarely, some minor complications could be detected.

## Data Availability

The datasets used and analysed during the current study are available from the corresponding author on reasonable request.

## Abbreviations

APT: Active periodontal therapy
BOP: Bleeding on probing
PPD: Periodontal pocket depth
CAL-V: Vertical attachment level
SUP: Suppuration
mGI: Modified gingival index
mPII: Modified plaque index
SD: Standard deviation
FDP: Fixed dental prosthesis
